# Supplementary material for: Impact of number and type of identified antigen on transplant-free survival in hypersensitivity pneumonitis
Source: PLoS One. 2022 Sep 1;17(9):e0273544. doi: 10.1371/journal.pone.0273544 (PMC9436128; doi:10.1371/journal.pone.0273544)
Supplement: Attachment — Submitted filename: Response to reviewers 2.docx [file pone.0273544.s003.docx]

Response to reviewers

**Reviewer #1: The authors have addressed all of this reviewer's concerns. The manuscript is greatly improved. Great job!**

We appreciate the reviewer feedback.

**Reviewer #2: The manuscript is now more clear. The response is satisfactory. Just a few remaining points.
1) Your response indicates that all patients included in the study had a BAL, TBBx, and/or SLB. I assume there were patients with BAL but no Tbbx? If so, please update Table 1 to include total number of patients with BAL (with or without Tbbx) in each column since BAL is a separate criterion in the diagnostic algorithm.**

We have added the total number of patients with BAL (N=57, 49 of whom also had TBBx) to table 1 as requested.

We also created a note underneath the table with the following text to improve transparency with regard to diagnostic workup in our cohort.

***30 patients had both SLB and Tbbx

****49 patients had both BAL and TBBx

**2) Please add IRB approval protocol number**

We have added the IRB approval number.

“This study was approved by the Institutional Review Board at University of Texas Southwestern Medical Center (IRB approval protocol number STU-2021-0598), and consent was waived for the study.”
